# Systemic Comorbidities in Korean Patients with Rosacea: Results from a Multi-Institutional Case-Control Study

**DOI:** 10.3390/jcm9103336

**Published:** 2020-10-17

**Authors:** Yu Ri Woo, Hei Sung Kim, Se Hoon Lee, Hyun Jeong Ju, Jung Min Bae, Sang Hyun Cho, Jeong Deuk Lee

**Affiliations:** 1 Department of Dermatology, Incheon St. Mary’s Hospital, The Catholic University of Korea, Seoul 06591, Korea; w1206@naver.com (Y.R.W.); hazelkimhoho@gmail.com (H.S.K.); leesehoon92@gmail.com (S.H.L.); drchos@yahoo.co.kr (S.H.C.); 2 Dr. Philip Frost Department of Dermatology and Cutaneous Surgery, Miami Itch Center, Miller School of Medicine, University of Miami, Miami, FL 33136, USA; 3 Department of Dermatology, St. Vincent’s Hospital, The Catholic University of Korea, Seoul 06591, Korea; hyd0116@naver.com (H.J.J.); jiminbae@gmail.com (J.M.B.); 4 Heal House Skin Clinic, Mesanro 24, Paldal-gu, Suwon 16461, Korea

**Keywords:** systemic comorbidities, Korea, rosacea, multi-institutional case-control study

## Abstract

Recent evidence links rosacea to systemic disease, but there are not enough methodologic studies addressing this association in Asians. Our aim was to identify rosacea comorbidities in Koreans and establish a reference database. A multi-center, case-control study was performed where a total of 12,936 rosacea patients and 12,936 age- and sex-matched control subjects were identified from 2007 to 2018. Logistic regression was performed to find significant association between rosacea and Sjögren syndrome (odds ratio [OR] 2.05; 95% confidence interval, 1.40–3.00), systemic sclerosis (OR 6.56; 95% CI, 1.50–28.7), rheumatoid arthritis (OR 1.72; 95% CI, 1.50–1.98), ankylosing spondylitis (OR 2.32; 95% CI, 1.42–3.84), autoimmune thyroiditis (OR 1.96; 95% CI, 1.40–2.73), alopecia areata (OR 1.77; 95% CI, 1.27–2.45), vitiligo (OR 1.90; 95% CI, 1.30–2.77), lung cancer (OR 1.54; 95% CI, 1.06–2.21), hepatobiliary cancer (OR 1.38; 95% CI, 1.06–1.77), alcohol abuse (OR 1.59; 95% CI, 1.05–2.39), diabetes mellitus (OR 1.11; 95% 1.02–1.19), obesity (OR 1.72; 95% CI, 1.22–2.41), allergic rhinitis (OR 1.65; 95% CI, 1.54–1.76), allergic conjunctivitis (OR 1.57; 95% CI, 1.27–1.94), chronic rhinosinusitis (OR 1.28; 95% CI, 1.14–1.42), herpes infection (OR 1.69; 95% CI, 1.53–1.86), and human papillomavirus infection (OR 2.50; 95% CI, 2.06–3.02). Higher odds for Sjogren syndrome, systemic sclerosis, ankylosing spondylitis, thyroiditis, vitiligo, hepatobiliary cancer, and obesity was exclusive in female subjects with rosacea, whereas increased prevalence of alopecia areata and alcohol abuse was confined to men. Only those who were 50 years and older exhibited higher odds for vitiligo, lung cancer, and gastroesophageal reflux disease while individuals younger than 50 were exclusively associated with hepatobiliary cancer, allergic conjunctivitis, and irritable bowel syndrome. Our study suggests that Koreans with rosacea are more likely to experience systemic comorbidity. Clinicians should acknowledge these interrelations and employ comprehensive care with an individual-based approach.

## 1. Introduction

Rosacea is a chronic relapsing dermatosis that affects millions throughout the world (prevalence ranging from 2% to 22% in the US and Europe, respectively) [1,2]. It is clinically diagnosed based on the presence of facial phyma, persistent erythema (diagnostic features), flushing/transient erythema, papules and pustules, telangiectasia (major features), skin burning, stinging, dryness, and edema (minor features) [3,4]. While the exact pathophysiology of rosacea remains unclear, it is generally accepted as an inflammatory disease [5], where the complex interplay between genetic and environmental factors is thought to alter the immune function, triggering chronic skin inflammation [6,7,8,9].

Once believed a disorder confined to the skin, now there is mounting evidence linking rosacea with systemic illnesses. In recent studies, rosacea patients were shown to carry greater risk of cardiovascular diseases, allergies, psychiatric problems, gastrointestinal disorders, malignancies, and autoimmune conditions relative to controls without rosacea [10,11,12,13,14,15,16,17,18].

While rosacea is thought to be most common in fair-skinned individuals, it may simply be more difficult to diagnose in those with skin of color. Rosacea has in fact been reported in Africa and Asia whose populations consist significant proportions of people with skin of color with rates up to 10% [19,20].

Due to the lower index of interest for rosacea among patients with darker skin, data on disease associated with rosacea in Asians are still relatively sparse and not thoroughly evaluated [5,21,22]. The aim of this study was to investigate the odds of systemic comorbidities in Korean patients with rosacea. In addition, we analyzed the effects of age and sex on the associations between rosacea and various systemic disorders.

South Korea employs a mandatory National health Insurance (NHI) system that enables nationwide population-based studies. Unfortunately, a number of dermatologic disorders labeled as “cosmetic disorders” (i.e., rosacea, acne, melasma, androgenetic alopecia) are not subsidized and are largely absent from the NHI database. In light of this matter, our study was conducted based on a multi-institutional hospital database.

## 2. Materials and Methods

### 2.1. Data Source

We performed a multi-institutional cross-sectional study by obtaining the electronic medical records (EMR) data from seven affiliated hospitals of the Catholic University of Korea (Incehon, Seoul, Yeouido, Uijeongbu, Bucheon, Eunpyeong St. Mary’s hospital and St. Vincent’s Hospital). The hospital database (nU system) encompasses all subsidized and non-subsidized claims, where the diagnostic codes are in the format of the International Classification of Disease, Tenth revision, Clinical Modification (ICD-10). The data includes details regarding patient age, sex, diagnosis, prescriptions, medical speciality one reached out for, and dates of outpatient visits.

### 2.2. Ethics

This study was reviewed and approved by the Catholic University of Korea Ethics Committee (XC19REDI0064) and was conducted according to the principles of the Declaration of Helsinki.

### 2.3. Study Population

All individuals with a primary diagnosis of rosacea including rhinophyma, other rosacea, and rosacea unspecified (*ICD-10-CM* codes L71, L71.1, L71.8, L71.9) between 2007 and 2018 were extracted from our hospital database. To ensure diagnostic validity, we limited our subjects to those who made at least two visits to the dermatology outpatient clinic under the diagnosis. From the population, we removed patients who did not receive any therapeutic intervention (i.e., prescription medication) for rosacea and those who claimed twice or more to have acne (*ICD-10-CM* code L70), seborrheic dermatitis (*ICD-10-CM* code L21), or cutaneous and systemic lupus erythematosus (*ICD-10-CM* code L93 and M32 respectively). The control group was selected randomly from age- and sex-matched individuals without rosacea who had undergone hemorrhoidectomy or appendectomy within the same time-period (between 2007 and 2018).

### 2.4. Concurrent Diseases

The outcomes of interest were concurrent systemic diseases including autoimmune disease (Sjogren syndrome, systemic sclerosis, Bechet’s disease, rheumatoid arthritis, ankylosing spondylitis, autoimmune thyroiditis, alopecia areata, and vitiligo), cancer (nonmelanoma skin cancer, melanoma, thyroid cancer, lung cancer, gastrointestinal cancer, and hepatobiliary cancer), neurologic disorder (Parkinson’s disease, dementia, and migraine), mental disorder (depression, anxiety disorder, and alcohol abuse), cardiovascular disorder (hypertension and coronary heart disease), metabolic disorder (diabetes mellitus, obesity, and dyslipidemia), allergic disorder (allergic rhinitis, allergic conjunctivitis, and asthma), respiratory disorder (chronic rhinosinusitis and chronic obstructive pulmonary disorder), gastrointestinal disorder (gastroesophageal reflux disease, helicobacter pylori infection, and irritable bowel syndrome), and infectious disorder (chronic viral hepatitis, tuberculosis, herpes infection, and human papillomavirus infection). To be designated as having a certain disease, the patient had to have at least two diagnoses made by a physician with the corresponding specialty during the study period (2007 to 2018). The diagnostic code for each comorbidity is summarized in Supplementary Table S1.

### 2.5. Statistical Analysis

We calculated adjusted odds ratios (ORs) and 95% confidence intervals (CIs) using conditional logistic regression modeling. We performed subsequent subgroup analysis in terms of sex and age. All statistical analysis was performed using IBM SPSS version 26.0 (SPSS Inc., Chicago, IL, USA). *p*-value of less than 0.05 was considered statistically significant.

## 3. Results

### 3.1. Demographic Characteristics of Rosacea Patients and Control Subjects

We identified a total of 12,936 rosacea patients and 12,936 control subjects without rosacea. The mean subject age was 47.4 ± 0.13 years, and 66.0% of subjects were female. The baseline characteristics of the study population are presented in Table 1.

### 3.2. Association between Rosacea and Systemic Diseases

When analyzed by conditional logistic regression for matched case-control pairs, patients with rosacea were significantly associated with a number of autoimmune disease [Sjögren syndrome (aOR*, 2.05; 95% CI, 1.40–3.00; *p* < 0.001), systemic sclerosis (aOR*, 6.57; 95% CI, 1.50–28.7; *p* = 0.012), rheumatoid arthritis (aOR*, 1.72; 95% CI, 1.50–1.98; *p* < 0.001), ankylosing spondylitis (aOR*, 2.34; 95% CI, 1.42–3.84; *p* = 0.001), autoimmune thyroiditis (aOR*, 1.96; 95% CI, 1.40–2.73; *p* < 0.001), alopecia areata (aOR*, 1.77; 95% CI, 1.27–2.45; *p* = 0.001), and vitiligo (aOR*, 1.90; 95% CI, 1.30–2.77; *p* = 0.001)], cancer [lung cancer (aOR*, 1.54; 95% CI, 1.06–2.21; *p* = 0.021) and hepatobiliary cancer (aOR*, 1.38; 95% CI, 1.06–1.77; *p* = 0.014)], mental disorder [alcohol abuse (aOR*, 1.59; 95% CI, 1.05–2.39; *p* = 0.027)], metabolic disorder [type 2 diabetes mellitus (aOR*, 1.11; 95% CI, 1.02–1.19; *p* = 0.010) and obesity (aOR*, 1.72; 95% CI, 1.22–2.41; *p* = 0.002)], allergic disease [allergic rhinitis (aOR*, 1.65; 95% CI, 1.54–1.76; *p* < 0.021) and allergic conjunctivitis (aOR*, 1.57; 95% CI, 1.27–1.94; *p* < 0.021)], respiratory disease [chronic rhinosinusitis (aOR*, 1.28; 95% CI, 1.14–1.42; *p* < 0.001)], and infectious disorder [herpes infection (aOR*, 1.69; 95% CI, 1.53–1.86; *p* < 0.001) and HPV infection (aOR*, 2.50; 95% CI, 2.06–3.02; *p* < 0.001)] (Table 2).

### 3.3. Subgroup Analysis According to Sex

Upon subgroup analysis by sex, female patients with rosacea showed significant association with a variety of autoimmune disease [Sjögren syndrome (aOR*, 2.10; 95% CI, 1.41–3.11; *p* < 0.001), systemic sclerosis (aOR*, 6.18; 95% CI, 1.40–27.2; *p* = 0.016), rheumatoid arthritis (aOR*, 1.78; 95% CI, 1.52–2.07; *p* < 0.001), ankylosing spondylitis (aOR*, 2.09; 95% CI, 1.11–3.92; *p* = 0.022), autoimmune thyroiditis (aOR*, 1.91; 95% CI, 1.34–2.71; *p* < 0.001), and vitiligo (aOR*, 1.86; 95% CI, 1.21–2.85; *p* = 0.004)], cancer [hepatobiliary cancer (aOR*, 1.47; 95% CI, 1.01–2.14; *p* = 0.042)], metabolic disorder [obesity (aOR*, 1.70; 95% CI, 1.18–2.43; *p* = 0.004)], allergic disease [allergic rhinitis (aOR*, 1.62; 95% CI, 1.48–1.75; *p* < 0.001) and allergic conjunctivitis (aOR*, 1.52; 95% CI, 1.20–1.93; *p* < 0.001)], respiratory disease [chronic rhinosinusitis (aOR*, 1.29; 95% CI, 1.13–1.47; *p* < 0.001)], and infectious disorder [herpes infection (aOR*, 1.60; 95% CI, 1.42–1.79; *p* < 0.001) and HPV infection (aOR*, 2.25; 95% CI, 1.78–2.81; *p* < 0.001)] (Table 3).

Male subjects with rosacea were significantly associated with a number of autoimmune diseases [rheumatoid arthritis (aOR*, 1.53; 95% CI, 1.11–2.08; *p* = 0.008), ankylosing spondylitis (aOR*, 0.98; 95% CI, 0.96–0.99; *p* = 0.013), and alopecia areata (aOR*, 3.96; 95% CI, 1.64–9.51; *p* = 0.002)], mental disorder [alcohol abuse (aOR*, 1.94; 95% CI, 1.15–3.24; *p* = 0.012)], allergic disease [allergic rhinitis (aOR*, 1.74; 95% CI, 1.52–1.97; *p* < 0.001) and allergic conjunctivitis (aOR*, 1.75; 95% CI, 1.07–2.86; *p* = 0.025)], respiratory disease [chronic rhinosinusitis (aOR*, 1.24; 95% CI, 1.10–1.52; *p* = 0.043)], and infectious disorder [herpes infection (aOR*, 1.99; 95% CI, 1.62–2.44; *p* < 0.001) and HPV infection (aOR*, 3.15; 95% CI, 2.20–4.51; *p* < 0.001)] (Table 3).

### 3.4. Subgroup Analysis According to Age

When stratified with age, rosacea patients 50 years and older were significantly associated with a number of autoimmune disease [Sjögren syndrome (aOR*, 1.91; 95% CI, 1.21–3.01; *p* = 0.005), systemic sclerosis (aOR*, 8.44; 95% CI, 1.07–66.4; *p* = 0.04), rheumatoid arthritis (aOR*, 1.69; 95% CI, 1.42–2.01; *p* < 0.001), ankylosing spondylitis (aOR*, 2.51; 95% CI, 1.25–5.00; *p* = 0.009), autoimmune thyroiditis (aOR*, 1.92; 95% CI, 1.27–2.89; *p* = 0.002), alopecia areata (aOR*, 2.00; 95% CI, 1.19–3.34; *p* = 0.008), and vitiligo (aOR*, 1.96; 95% CI, 1.26–3.01; *p* = 0.002)], cancer [lung cancer (aOR*, 1.53; 95% CI, 1.04–2.25; *p* = 0.030)], metabolic disorder [obesity (aOR*, 2.00; 95% CI, 1.17–3.40; *p* = 0.002)], allergic disease [allergic rhinitis (aOR*, 1.59; 95% CI, 1.45–1.72; *p* < 0.001)], respiratory disease [chronic rhinosinusitis (aOR*, 1.23; 95% CI, 1.06–1.41; *p* = 0.005)], gastrointestinal disorder [gastroesophageal reflux disease (GERD) (aOR*, 1.54; 95% CI, 1.10–2.07; *p* = 0.001)], and infectious disorder [herpes infection (aOR*, 1.64; 95% CI, 1.45–1.85; *p* < 0.001) and HPV infection (aOR*, 2.47; 95% CI, 1.84–3.29; *p* < 0.001)] (Table 3).

Subjects under 50 years of age showed association with a variety of autoimmune diseases [Sjögren syndrome (aOR*, 2.45; 95% CI, 1.17–5.06; *p* = 0.016), rheumatoid arthritis (aOR*, 1.69; 95% CI, 1.33–2.14; *p* < 0.001), ankylosing spondylitis (aOR*, 2.14; 95% CI, 1.08–4.24; *p* = 0.029), autoimmune thyroiditis (aOR*, 2.01; 95% CI, 1.13–3.56; *p* = 0.017), and alopecia areata (aOR*, 1.70; 95% CI, 1.11–2.59; *p* = 0.013)], cancer [hepatobiliary cancer (aOR*, 2.27; 95% CI, 1.25–4.11; *p* = 0.007)], metabolic disorders [obesity (aOR*, 1.58; 95% CI, 1.03–2.39; *p* = 0.033)], allergic diseases [allergic rhinitis (aOR*, 1.79; 95% CI, 1.59–2.01; *p* < 0.001) and allergic conjunctivitis (aOR*, 2.51; 95% CI, 1.70–3.70; *p* = 0.001)], respiratory disease [chronic rhinosinusitis (aOR*, 1.40; 95% CI, 1.17–1.67; *p* < 0.001)], gastrointestinal disorder [irritable bowel syndrome (aOR*, 1.43; 95% CI, 1.51–1.97; *p* < 0.001], and infectious disorders [herpes infection (aOR*, 1.83; 95% CI, 1.54–2.17; *p* < 0.001) and HPV infection (aOR*, 3.24; 95% CI, 2.49–4.21; *p* < 0.001)] (Table 3).

## 4. Discussion

With a growing body of literature linking rosacea to systemic diseases, there is definite need for further exploration. So far, many of the larger-scale epidemiologic studies on rosacea comorbidity have been restricted to Caucasians, resulting in limited generalizability of the findings.

In this study, we found substantially higher odds for a number of autoimmune disease, cancer (lung cancer and hepatobiliary cancer), alcohol abuse, metabolic disorder (type 2 diabetes mellitus and obesity), allergic disease (allergic rhinitis and allergic conjunctivitis), chronic rhinosinusitis, and infectious disorder (herpes infection and human papillomavirus infection) among Korean rosacea patients seen in secondary/tertiary medical centers.

The association between autoimmune disorder and rosacea has not been studied well and intrigued us the most. While Egeberg et al. [17] have reported that European subjects with rosacea have increased ORs for type 1 diabetes mellitus, celiac disease, multiple sclerosis, and rheumatoid arthritis, we did not look into the first three diseases because they are extremely rare in Koreans. We did examine a variety of autoimmune disorders to find strong association between rosacea and Sjögren syndrome, systemic sclerosis, rheumatoid arthritis, ankylosing spondylitis, autoimmune thyroiditis, alopecia areata, and vitiligo among Koreans. Notably, greater odds for Sjogren syndrome, systemic sclerosis, ankylosing spondylitis, thyroiditis, and vitiligo were exclusive to female subjects with rosacea, whereas a heightened prevalence of alopecia areata was confined to men. Interestingly, only those who were 50 years and older exhibited greater odds for vitiligo. Although no rosacea-specific autoantibodies have been identified, the increased risk of autoimmune diseases in rosacea, especially in female and the elderly who show higher frequency of autoantibodies, suggest their involvement in rosacea [23]. A genome-wide association study on Europeans revealed a link between rosacea and human leukocyte antigen (HLA) alleles, which are also connected with autoimmune diseases [16,24]. Another explanation to this interrelation may be that both entities share common inflammatory elements. Upregulation of interleukin (IL)-1 [25,26,27,28,29,30], interferon (IFN)-γ [31,32,33,34], and toll-like receptors (TLRs) [35,36] has been observed in both rosacea and autoimmune diseases.

Few studies have analyzed the connection between cancer and rosacea. A Danish group found that individuals with rosacea more often develop breast cancer, non-melanoma skin cancer (NMSC), and hepatic cancer [37]. In a US study [38], women with a personal history of rosacea showed an increased risk of incident basal cell carcinoma and thyroid cancer. Unlike these two studies, a Taiwanese study failed to find any association between rosacea and cancer (including skin cancer) [39]. As for our findings, we also observed no interrelation between rosacea and skin cancer. Although Koreans with rosacea tend to have lighter complexion, Asian ethnicity seems to attenuate the impact of rosacea on skin cancer [40]. Notably, hepatobiliary cancer was more common in Koreans with rosacea, which is in accordance with results from the Danish study [37]. The higher odds for alcohol consumption in our patients may explain this outcome as there is causal association between alcohol intake and hepatobiliary cancer. Both hepatobiliary cancer and rosacea overexpress vascular growth factors (i.e., vascular endothelial growth factor, fibroblast growth factor) [41,42,43,44], which may also account for the observed connection.

Alcohol consumption can induce blood vessel dilatation and inflammation and has been identified as a risk factor for rosacea [45,46]. Our study confirmed the association between rosacea and alcohol abuse, and should be inquired, especially in men.

A strong association between rosacea and metabolic disorder (i.e., diabetes mellitus and obesity) has been recognized in prior studies [47,48] and ours. It is postulated that systemic inflammation underlying rosacea induces structural changes of the lipoprotein, which adversely affects the lipid profile [49,50]. Low serum activity of paraoxonase-1 (PON-1), an antioxidant enzyme that prevents oxidation of serum lipoprotein, is a shared feature between rosacea [5,51] and metabolic disease [52] and suggests that oxidative stress contributes to their co-occurrence. Obesity was more prominent in Korean women, which should be given consideration in management.

With regards to allergic disease, Rainer et al. [18]. claimed that patients with rosacea are more likely to experience food and airborne allergies. In this study, we analyzed the presence of various allergies in rosacea patients to find higher odds of allergic rhinitis and allergic conjunctivitis in Koreans with rosacea. Further large-scale, long-term study would be needed to confirm the possible association between atopic dermatitis and rosacea.

Interestingly, we observed a strong association between skin/mucosal infection (herpes and human papillomavirus infection) and rosacea in Koreans, which may owe to the disrupted skin barrier and skewed immunity. Conversely, we found no connection between rosacea and chronic systemic infection (chronic viral hepatitis and tuberculosis).

The association between cardiovascular conditions and rosacea is still controversial [5,52,53,54]. In a 2016 study, Egeberg et al. [53] reported that the cardiovascular risk in Danish patients with rosacea is generally comparable with control subjects albeit with a slightly lower risk of myocardial infarction (after adjustment of confounding factors), which is consistent with our findings. Since we identified rosacea and cardiovascular conditions based on secondary/tertiary hospital recordings, it may only represent a subgroup of patients and should be interpreted accordingly. Interestingly, a recent publication has shown that tetracycline treatment in rosacea reduces the risk of vascular events [55,56]. Also, certain treatment for cardiovascular disease (i.e., beta-blockers for hypertension) attenuate rosacea, which may have contributed to the outcome.

To our surprise, we were not able to find any significant association between rosacea and gastrointestinal, neurologic and mental diseases, which is in sharp contrast with the previous literature [57,58,59]. Ethnic difference in the use of mental health service and disease symptom may account for this disparity, which needs further research [60,61,62,63,64,65].

The present study has limitations. First, as we utilized the hospital database, we were not able to identify rosacea subtype and disease severity, as well as the lifestyle risk factors. The influence of these factors should be evaluated in future studies to validate our findings. Second, the health care setting we used may have affected the likelihood of comorbid disease. Another limitation is the lack of a clear temporal relationship between the occurrence of rosacea and the comorbid diseases, due to the retrospective character of the study. It is necessary to find the common pathophysiology between rosacea and the systemic diseases to determine if comorbidity associations are causal.

In conclusion, our study is the very first to assess rosacea comorbidities in the Korean population. We identified a high comorbidity burden where more of the rosacea patients had autoimmune disease (Sjögren syndrome, systemic sclerosis, rheumatoid arthritis, ankylosing spondylitis, autoimmune thyroiditis, alopecia areata, and vitiligo), cancer (lung cancer and hepatobiliary cancer), alcohol abuse, metabolic disorder (type 2 diabetes mellitus and obesity), allergic disease (allergic rhinitis and allergic conjunctivitis), chronic rhinosinusitis, and infectious disorder (herpes infection and human papillomavirus infection). Notably, there were higher odds for alcohol abuse in males and obesity in females when stratified by sex. The findings advise that clinicians be aware of these comorbidities and carefully assess Korean rosacea patients. Lastly, further genetic, epidemiological, and clinical studies on rosacea and its comorbidities over different ethnicities are required to gain a generalized understanding of rosacea.

## Figures and Tables

**Table 1:** Characteristics of the study population.

	Patients with Rosacea (*n* = 12,936)	Controls without Rosacea (*n* = 12,936)
Sex, *n* (%)		
Female	8540 (66.0)	8540 (66.0)
Male	4396 (34.0)	4396 (34.0)
Age, years, *n* (%)	47.4 ± 0.13	48.4 ± 0.13
<20	679 (5.3)	679 (5.3)
20–39	3363 (26.0)	3363 (26.0)
40–59	6252 (48.3)	6252 (48.3)
60–79	2538 (19.6)	2538 (19.6)
>80	104 (0.80)	104 (0.80)

Data are presented as number (%).

**Table 2:** Association between rosacea and various systemic diseases.

Variables	Number of Cases		OR	95% CI	*p*-Value	aOR*	95% CI	*p*-Value
	Rosacea	Control						
Autoimmune Disorder								
Sjögren syndrome	94	37	1.90	1.29–2.77	<0.001	2.05	1.40–3.00	<0.001
Systemic sclerosis	17	2	6.41	1.42–26.5	<0.001	6.57	1.50–28.7	0.012
Behcet’s disease	47	29	1.21	0.76–1.94	0.399	1.16	0.72–1.87	0.540
Rheumatoid arthritis	596	272	1.64	1.42–1.88	<0.001	1.72	1.5–1.98	<0.001
Ankylosing spondylitis	72	21	2.55	1.56–4.15	<0.001	2.34	1.42–3.84	0.001
Autoimmune thyroiditis	121	49	1.85	1.32–2.57	<0.001	1.96	1.40–2.73	<0.001
Alopecia areata	133	49	2.02	1.45–2.79	<0.001	1.77	1.27–2.45	0.001
Vitiligo	97	40	1.83	1.26–2.65	0.001	1.90	1.30–2.77	0.001
Cancer								
NMSC	43	26	1.28	0.78–2.07	0.329	1.52	0.94–2.46	0.086
Melanoma	10	6	1.26	0.44–3.58	0.670	1.43	0.48–4.22	0.514
Thyroid cancer	130	111	1.08	0.68–1.34	0.330	1.01	0.70–1.38	0.477
Lung cancer	84	46	1.34	0.93–1.93	0.111	1.54	1.06–2.21	0.021
Gastrointestinal cancer	265	326	0.74	0.67–0.83	0.230	0.79	0.60–0.78	0.270
Hepatobiliary cancer	157	89	1.32	1.02–1.71	0.034	1.38	1.06–1.77	0.014
Neurologic disorder								
Parkinson’s disease	98	90	1.01	0.60–1.28	0.154	1.04	0.70–1.35	0.669
Dementia	168	197	0.64	0.52–0.78	0.042	0.80	0.65–0.97	0.051
Migraine	54	58	0.70	0.48–1.01	0.060	0.66	0.45–0.96	0.053
Mental Disorder								
Depression	890	691	1.01	0.88–1.05	<0.001	1.03	0.93–1.12	<0.001
Anxiety disorder	757	582	1.14	0.76–1.72	<0.001	1.03	0.92–1.13	<0.001
Alcohol abuse	79	34	1.75	1.16–2.62	0.007	1.59	1.05–2.39	0.027
Cardiovascular Disorder								
Hypertension	1541	1527	1.02	0.70–1.39	0.051	1.02	0.78–1.22	0.062
Coronary heart disease	710	813	0.75	0.70–0.79	0.201	0.83	0.78–0.88	0.120
Metabolic Disorder								
Diabetes mellitus	1290	950	1.08	0.91–1.16	0.643	1.11	1.02–1.19	0.010
Obesity	121	49	1.86	1.33–2.59	<0.001	1.72	1.22–2.41	0.002
Dyslipidemia	1857	1594	1.07	0.81–1.21	<0.001	1.12	0.84–1.36	0.001
Allergic Disorder								
Allergic rhinitis	2064	938	1.68	1.57–1.80	<0.001	1.65	1.54–1.76	<0.001
Allergic conjunctivitis	259	121	1.59	1.28–1.96	<0.001	1.57	1.27–1.94	<0.001
Asthma	443	356	1.04	0.81–1.16	0.324	1.05	0.83–1.19	0.471
Respiratory Disorder								
Chronic rhinosinusitis	781	449	1.31	1.17–1.45	<0.001	1.28	1.14–1.42	<0.001
COPD	190	222	0.64	0.53–0.77	0.063	0.72	0.6–0.87	0.053
Gastrointestinal Disorder								
GERD	3118	2487	1.04	0.89–1.21	0.053	1.05	0.91–1.19	0.052
*H. pylori* infection	56	34	1.14	0.75–1.73	0.536	1.14	0.74–1.72	0.554
Irritable bowel syndrome	1472	1226	1.05	0.60–1.29	<0.001	1.18	0.62–1.42	<0.001
Infectious Disorder								
Chronic viral hepatitis	296	248	1.01	0.75-1.16	0.213	1.01	0.74–1.15	0.180
Tuberculosis	61	85	0.36	0.23-0.55	0.051	0.39	0.22–0.56	0.052
Herpes infection	1127	511	1.68	1.51-1.85	<0.001	1.69	1.53–1.86	<0.001
HPV infection	548	126	3.29	2.72-3.99	<0.001	2.50	2.06–3.02	<0.001

OR, odds ratio; CI, confidence interval; aOR*, adjusted odds ratio, adjusted by age and sex; NMSC, nonmelanoma skin cancer; COPD, chronic obstructive pulmonary disease; GERD, gastroesophageal reflux disease; HPV, human papillomavirus.

**Table 3:** Subgroup analyses of the association between rosacea and systemic diseases by sex and age.

Variables	Sex						Age					
	Male			Female			<50			≥50		
	aOR*	95% CI	*p*-Value	aOR*	95% CI	*p*-Value	aOR*	95% CI	*p*-Value	aOR*	95% CI	*p*-Value
Autoimmune disorder												
Sjogren syndrome	1.54	0.39–6.08	0.536	2.10	1.41–3.11	<0.001	2.45	1.17–5.06	0.016	1.91	1.21–3.01	0.005
Systemic sclerosis	N/A			6.18	1.40–27.2	0.016	N/A			8.44	1.07–66.4	0.04
Behcet’s disease	0.93	0.38–2.26	0.873	1.26	0.71–2.20	0.428	0.86	0.43–1.71	0.669	1.57	0.83–2.97	0.164
Rheumatoid arthritis	1.53	1.11–2.08	0.008	1.78	1.52–2.07	<0.001	1.69	1.33–2.14	<0.001	1.69	1.42–2.01	<0.001
Ankylosing spondylitis	0.98	0.96–0.99	0.013	2.09	1.11–3.92	0.022	2.14	1.08–4.24	0.029	2.51	1.25–5.00	0.009
Thyroiditis	2.50	0.87–7.18	0.088	1.91	1.34–2.71	<0.001	2.01	1.13–3.56	0.017	1.92	1.27–2.89	0.002
Alopecia areata	3.96	1.64–9.51	0.002	1.44	0.99–2.07	0.051	1.70	1.11–2.59	0.013	2.00	1.19–3.34	0.008
Vitiligo	2.03	0.89–4.56	0.089	1.86	1.21–2.85	0.004	1.95	0.92–4.10	0.077	1.96	1.26–3.01	0.002
Cancer												
NMSC	1.83	0.78–4.25	0.160	1.38	0.76–2.49	0.289	1.36	0.38–4.83	0.633	1.42	0.83–2.41	0.191
Melanoma	2.42	0.43–13.4	0.313	0.92	0.21–3.90	0.908	0.52	0.04–6.57	0.610	1.59	0.51–4.90	0.421
Thyroid cancer	1.04	0.18–1.19	0.078	1.07	0.73–1.36	1.802	1.02	0.54–1.34	0.354	1.09	0.71–1.48	0.962
Lung cancer	1.42	0.88–2.27	0.148	1.74	0.97–3.10	0.060	0.98	0.32–2.92	0.965	1.53	1.04–2.25	0.030
Gastrointestinal cancer	0.73	0.56–0.94	0.056	0.64	0.26–0.44	0.057	0.41	0.20–0.46	0.061	0.63	0.43–0.63	0.058
Hepatobiliary cancer	1.28	0.91–1.80	0.152	1.47	1.01–2.14	0.042	2.27	1.25–4.11	0.007	1.14	0.84–1.53	0.402
Neurologic Disorder												
Parkinson’s disease	1.16	0.72–1.88	0.535	0.84	0.58–1.20	0.332	1.03	0.25–1.10	0.089	1.09	0.72–1.35	0.956
Dementia	0.90	0.64–1.26	0.554	0.75	0.58–0.95	0.052	3.36	0.99–11.3	0.051	0.69	0.56–0.85	0.651
Migraine	0.83	0.38–1.79	0.629	0.61	0.39–0.95	0.059	0.56	0.32–0.95	0.054	0.80	0.47–1.33	0.385
Mental Disorder												
Depression	1.11	0.91–1.35	0.282	1.00	0.9–1.11	0.992	1.01	0.85–1.19	0.915	1.01	0.90–1.12	0.829
Anxiety disorder	1.13	0.91–1.38	0.262	0.99	0.88–1.11	0.906	1.07	0.89–1.28	0.449	0.98	0.86–1.10	0.699
Alcohol abuse	1.94	1.15–3.24	0.012	1.08	0.53–2.18	0.824	1.75	0.90–3.38	0.095	1.51	0.89–2.53	0.121
Cardiovascular Disorder												
Hypertension	1.05	0.86–1.25	0.332	1.06	0.70–1.22	0.532	1.03	0.71–1.25	0.051	1.01	0.74–1.15	0.053
Coronary heart disease	0.72	0.62–0.82	0.210	0.73	0.64–0.82	0.081	1.60	1.26–2.02	0.280	1.39	1.25–1.54	0.278
Metabolic Disorder												
Diabetes mellitus	1.12	0.98–1.26	0.076	1.10	0.99–1.21	0.063	1.12	0.94–1.32	0.186	1.06	0.97–1.15	0.159
Obesity	1.86	0.72–4.80	0.200	1.70	1.18–2.43	0.004	1.58	1.03–2.39	0.033	2.00	1.17–3.40	0.002
Dyslipidemia	1.22	0.82–1.45	0.054	1.21	0.78–1.44	0.068	1.01	0.70–1.22	0.066	1.20	0.72–1.43	0.051
Allergic Disorders												
Allergic rhinitis	1.74	1.52–1.97	<0.001	1.62	1.48–1.75	<0.001	1.79	1.59–2.01	<0.001	1.59	1.45–1.72	<0.001
Allergic conjunctivitis	1.75	1.07–2.86	0.025	1.52	1.20–1.93	<0.001	2.51	1.70–3.70	0.000	1.21	0.92–1.57	0.176
Asthma	0.89	0.69–1.13	0.35	0.98	0.83–1.15	0.793	0.99	0.78–1.25	0.924	0.94	0.79–1.10	0.425
Respiratory Disorders												
Chronic rhinosinusitis	1.24	1.00–1.52	0.043	1.29	1.13–1.47	<0.001	1.40	1.17–1.67	<0.001	1.23	1.06–1.41	0.005
COPD	0.91	0.71–1.15	0.438	0.52	0.37–0.70	0.051	0.48	0.28–0.81	0.057	0.73	0.59–0.89	0.052
Gastrointestinal disorders												
GERD	1.00	0.92–1.08	0.982	1.03	0.88–0.97	0.563	1.00	0.92–1.07	0.987	1.54	1.10–2.07	0.001
*H. pylori* infection	1.40	0.71–2.75	0.331	1.00	0.58–1.72	0.876	1.13	0.57–2.21	0.730	1.11	0.65–1.89	0.705
Irritable bowel syndrome	1.07	0.67–0.88	0.051	0.10	0.58–0.7	0.061	1.43	1.51–1.97	<0.001	0.72	0.65–0.78	0.063
Infectious disorder												
Chronic viral hepatitis	1.01	0.57–0.99	0.052	1.08	0.78–1.22	0.873	1.06	0.57–1.00	0.051	1.07	0.78–1.19	0.759
Tuberculosis	0.43	0.21–0.68	0.082	0.33	0.22–0.54	0.057	0.48	0.22–0.72	0.145	0.43	0.21–0.80	0.244
Herpes infection	1.99	1.62–2.44	<0.001	1.60	1.42–1.79	<0.001	1.83	1.54–2.17	<0.001	1.64	1.45–1.85	<0.001
HPV infection	3.15	2.20–4.51	<0.001	2.25	1.78–2.81	<0.001	3.24	2.49–4.21	<0.001	2.47	1.84–3.29	<0.001

aOR*, adjusted odds ratio, adjusted by age and sex; CI, confidence interval; NMSC, nonmelanoma skin cancer; COPD, chronic obstructive pulmonary disease; GERD, gastroesophageal reflux disease; HPV, human papillomavirus.

## Supplementary Materials

The following are available online at https://www.mdpi.com/2077-0383/9/10/3336/s1, Table S1: The ICD-10 (International Classification of Disease, 10th revision, Clinical Modification) codes used for each disease specification
