# Supplementary material for: Systemic Comorbidities in Korean Patients with Rosacea: Results from a Multi-Institutional Case-Control Study
Source: J Clin Med. 2020 Oct 17;9(10):3336. doi: 10.3390/jcm9103336 (PMC7603220; doi:10.3390/jcm9103336)
Supplement: Supplementary file 1 [file jcm-09-03336-s001.pdf]

Table S1: The ICD-10 (International Classification of Disease, 10th revision, Clinical Modification) codes used for each disease specification

	Disease	ICD-10 codes
Autoimmune disorders	Sjogren syndrome	M35.0
	Systemic sclerosis	M34
	Behcet's disease	M35.2
	Rheumatoid arthritis	M05-06
	Ankylosing spondylitis	M45
	Alopecia areata	L63
	Vitiligo	L80
	Autoimmune thyroiditis	E06.3
Cancer	Non melanoma skin cancer	C44
	Malignant melanoma	C43
	Thyroid cancer	C73
	Lung cancer	C34
	Gastrointestinal cancer	C16-C21
	Hepatobiliary cancer	C22-C25
Neurologic disorders	Parkinson's disease	G20-G22
	Dementia	G30, F00-F03
	Migraine	G43
Mental disorders	Depression	F32-33
	Anxiety disorder	F40-41
	Alcohol abuse	F10
Allergic disorders	Allergic rhinitis	J30.1-J30.4
	Allergic conjunctivitis	H10.1
	Asthma	J45-46
Respiratory disorders	Chronic rhinosinusitis	J32
	COPD	J44
Gastrointestinal disorders	GERD	K21
	H. pylori infection	B98.0
	Irritable bowel syndrome	K58
Cardiovascular disorders	Hypertension	I10
	Coronary heart disease	I24-25
Metabolic disorders	Diabetes mellitus	E11
	Obesity	E66
	Dyslipidemia or hyperlipidemia	E78
Infection	Chronic viral hepatitis	B18
	Tuberculosis	A15-19
	Herpes infection	A60, B00-02
	HPV infection	A63, B07, B97.7

COPD, chronic obstructive pulmonary disease; GERD, gastroesophageal reflux disease; HPV, human papillomavirus.
